# Combined Tibial Tubercle Fracture and Patellar Tendon Rupture in Adolescents: A Report of Three Cases Highlighting Fragment Rotation as a Diagnostic Clue

**DOI:** 10.7759/cureus.113277

**Published:** 2026-07-24

**Authors:** Javier Gonzalez Almonacid, Eduardo Poblete, Maximiliano Espinosa, Sergio Arellano, Rodrigo Donoso

**Affiliations:** 1 Knee Surgery, Hospital Clinico San Borja Arriaran, Santiago, CHL; 2 Orthopaedic Surgery, Clínica Alemana de Santiago, Santiago, CHL; 3 Orthopaedic Surgery, Universidad del Desarrollo, Santiago, CHL; 4 Orthopaedics, Clínica Alemana de Santiago, Santiago, CHL; 5 Orthopaedics and Trauma, Clínica Alemana de Santiago, Santiago, CHL; 6 Paediatric Orthopaedics, Hospital Roberto del Rio, Santiago, CHL

**Keywords:** adolescent knee injury, avulsion fracture, extensor mechanism injury, patellar tendon rupture, tibial tubercle fracture

## Abstract

We report three adolescent boys with combined tibial tubercle fracture and patellar tendon rupture. All presented with pain, swelling, and inability to actively extend the knee. Initial radiographs demonstrated tibial tubercle fractures, and complete fragment rotation was present in two cases. All patients underwent tibial tubercle fixation and patellar tendon repair. At final follow-up, all achieved fracture union, full active knee extension, and return to their pre-injury or higher activity level. Complete fragment rotation may represent an early radiographic clue to associated patellar tendon avulsion and may facilitate timely recognition and surgical treatment of this rare combined injury pattern.

## Introduction

Fractures of the tibial tubercle are uncommon in adolescents, accounting for less than 1% of physeal injuries. Most cases are isolated osseous avulsions, whereas associated patellar tendon rupture is much less common and has been described mainly in isolated case reports and small series [1,2].

Tibial tubercle fractures are commonly classified according to the Ogden classification, which is based on the location and extent of the fracture through the apophysis and proximal tibial physis. These injuries typically result from a sudden, forceful contraction of the quadriceps during jumping or landing activities in adolescents. Although most fractures occur as isolated injuries, the associated patellar tendon rupture may be overlooked during the initial assessment because of its rarity and the prominence of the osseous injury [1].

During skeletal maturation, the tibial tubercle apophysis and patellar tendon represent vulnerable transitional structures exposed to high tensile forces [3].

Failure to recognize the tendinous component at presentation may lead to delayed treatment and suboptimal restoration of the extensor mechanism [4]. We describe a multicenter series of adolescent patients with combined tibial tubercle fracture and patellar tendon rupture, with emphasis on radiographic findings, surgical management, and functional outcomes. We also highlight complete rotational displacement of the tibial tubercle fragment as a potential diagnostic clue to associated tendon injury [5].

Written informed consent for publication of anonymized clinical data and images was obtained from the patients' legal guardians.

## Case presentation

Case 1

**Figure 1:**
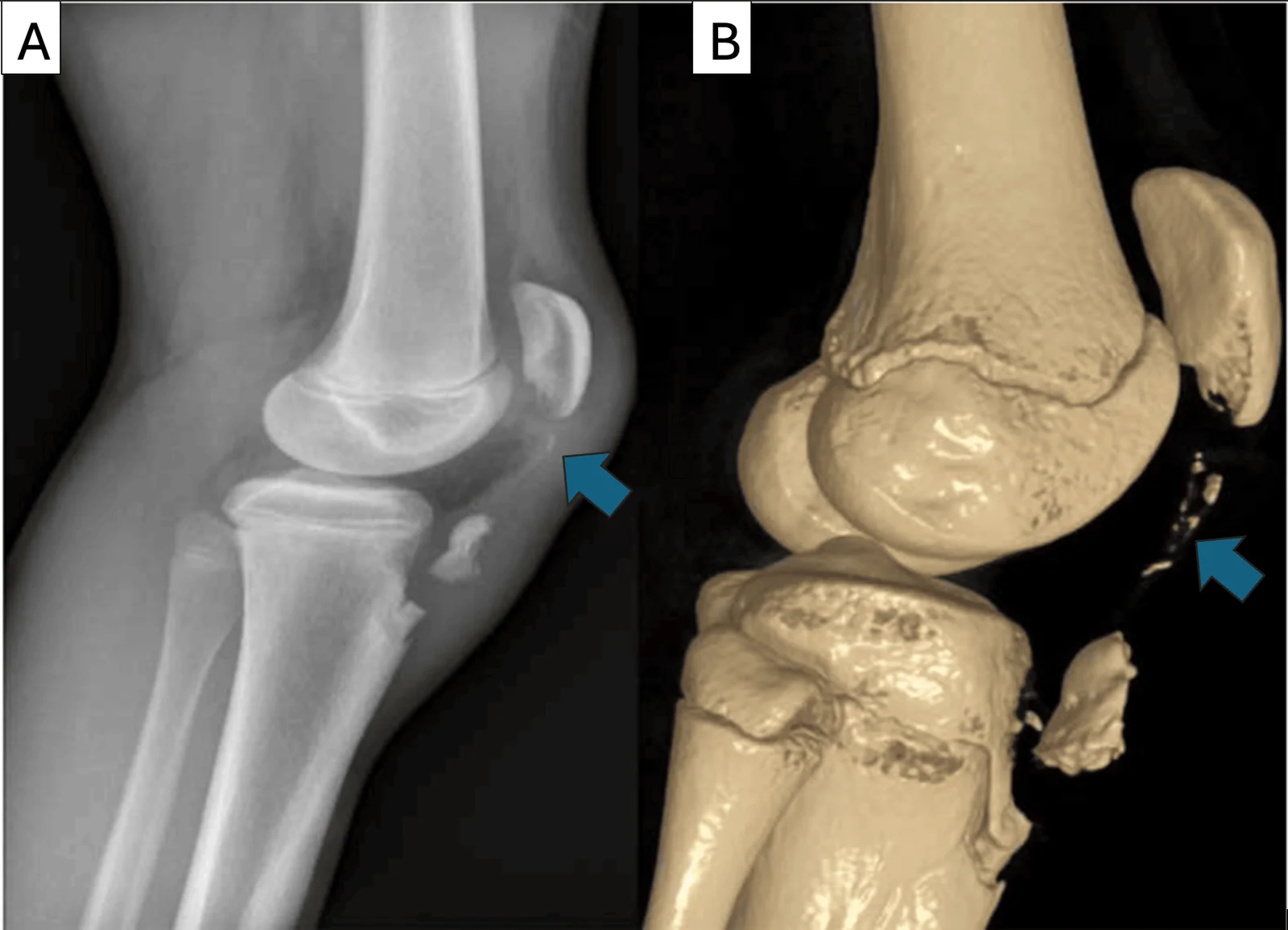
Preoperative lateral radiograph and three-dimensional computed tomography reconstruction of case 1. (A) Lateral radiograph demonstrating an Ogden type IIB tibial tubercle fracture with complete rotational displacement of the fragment. Small osseous fragments within the patellar tendon region (arrow) are consistent with the sign described by Kramer et al. [5] as a clue to associated patellar tendon avulsion. (B) Three-dimensional CT reconstruction confirming complete fragment rotation and the small osseous fragments within the patellar tendon region (arrow).

**Figure 2:**
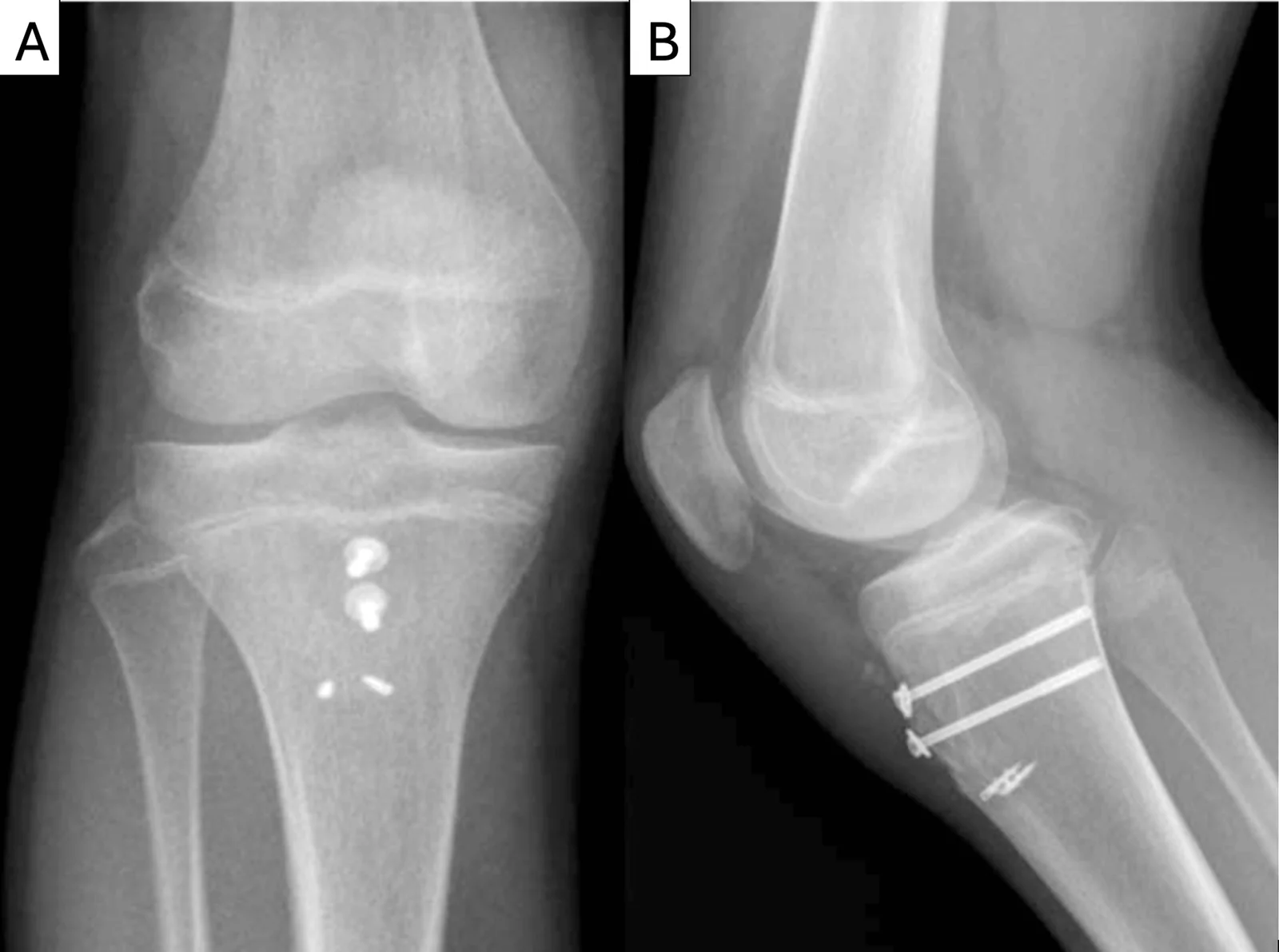
Postoperative anteroposterior and lateral radiographs of case 1. (A) Anteroposterior and (B) lateral radiographs obtained four months postoperatively, demonstrating maintained reduction, stable fixation, and fracture union following open reduction and internal fixation of the tibial tubercle with concomitant patellar tendon repair.

Patient 1 was a 13-year-old boy who sustained a right knee injury while jumping during rugby. He developed acute pain and functional incapacity immediately after the injury. Physical examination showed swelling over the tibial tubercle, patella alta, and inability to raise the leg with the knee extended. He had no relevant comorbidities or antecedent apophysitis. Radiographs and CT 3D reconstruction demonstrated an Ogden type IIB tibial tubercle fracture with complete rotational displacement of the fragment (Figure 1). The lesion consisted of an avulsion fracture of the tibial tubercle with fragment rotation and complete distal patellar tendon avulsion without an attached osseous fragment. He underwent open reduction and internal fixation with 3.5 mm Dart-Fire cannulated screws, and the tendon was repaired with two suture anchors and high-strength sutures using Krackow configuration. Fracture union was achieved by four months (Figure 2), and he returned to rugby by eight months. At final follow-up, 20 months after surgery, he had full active extension, no complications, a Lysholm score of 100, and a Tegner score of 9 both before injury and at final follow-up.

Case 2

**Figure 3:**
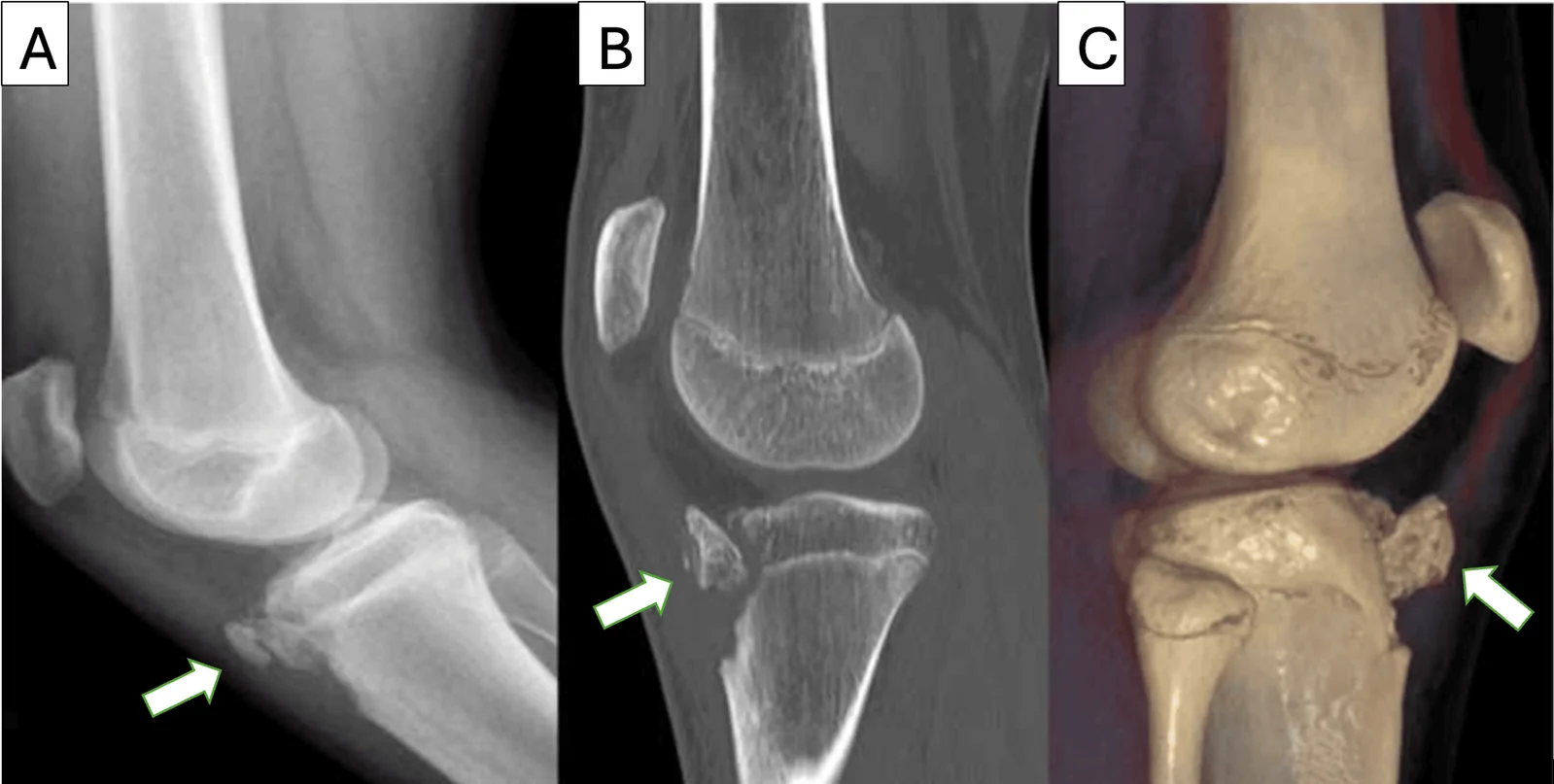
Preoperative imaging of case 2. (A) Lateral radiograph, (B) sagittal CT image, and (C) three-dimensional CT reconstruction demonstrating an Ogden type IB tibial tubercle fracture with complete rotational displacement of the fragment (arrows), suggestive of an associated patellar tendon avulsion.

**Figure 4:**
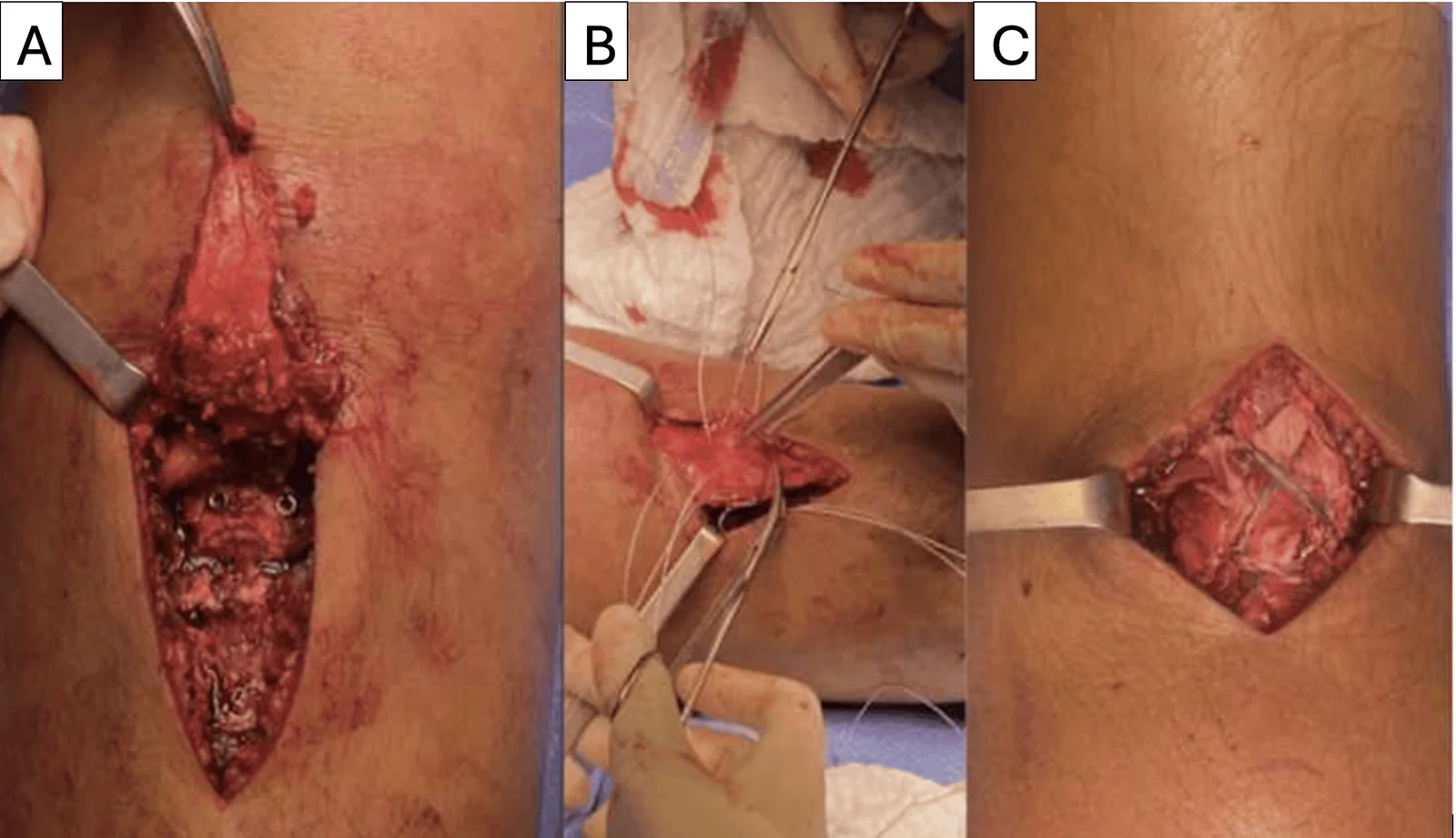
Intraoperative photographs of case 2. (A) Exposure of the bifocal extensor mechanism injury following reduction of the tibial tubercle fragment. (B) Patellar tendon repair using suture anchors and Krackow sutures. (C) Final construct after tibial tubercle fixation and patellar tendon repair.

Patient 2 was a 16-year-old boy who sustained a right knee injury during a jumping mechanism while playing sports. He presented with pain and inability to continue activity. On physical examination, the patient presented with knee swelling, inability to perform a straight-leg raise, loss of active knee extension, and a palpable defect over the patellar tendon. He had no comorbidities or antecedent apophysitis. Radiographs demonstrated an Ogden type IB tibial tubercle fracture with complete rotation of the fragment (Figure 3). The injury consisted of a tibial tubercle fracture with fragment rotation and complete distal patellar tendon avulsion. He underwent open reduction and internal fixation of the tibial tubercle, followed by patellar tendon repair with four suture anchors and Krackow sutures (Figure 4). Advanced fracture consolidation was documented by four months, and he returned to sports by seven months. At final follow-up, 27 months after surgery, he had full active extension and no complications. His Lysholm score was 89, and his Tegner score remained 8 before injury and at final follow-up. He returned to the same sport, although he still reported mild residual discomfort.

Case 3

**Figure 5:**
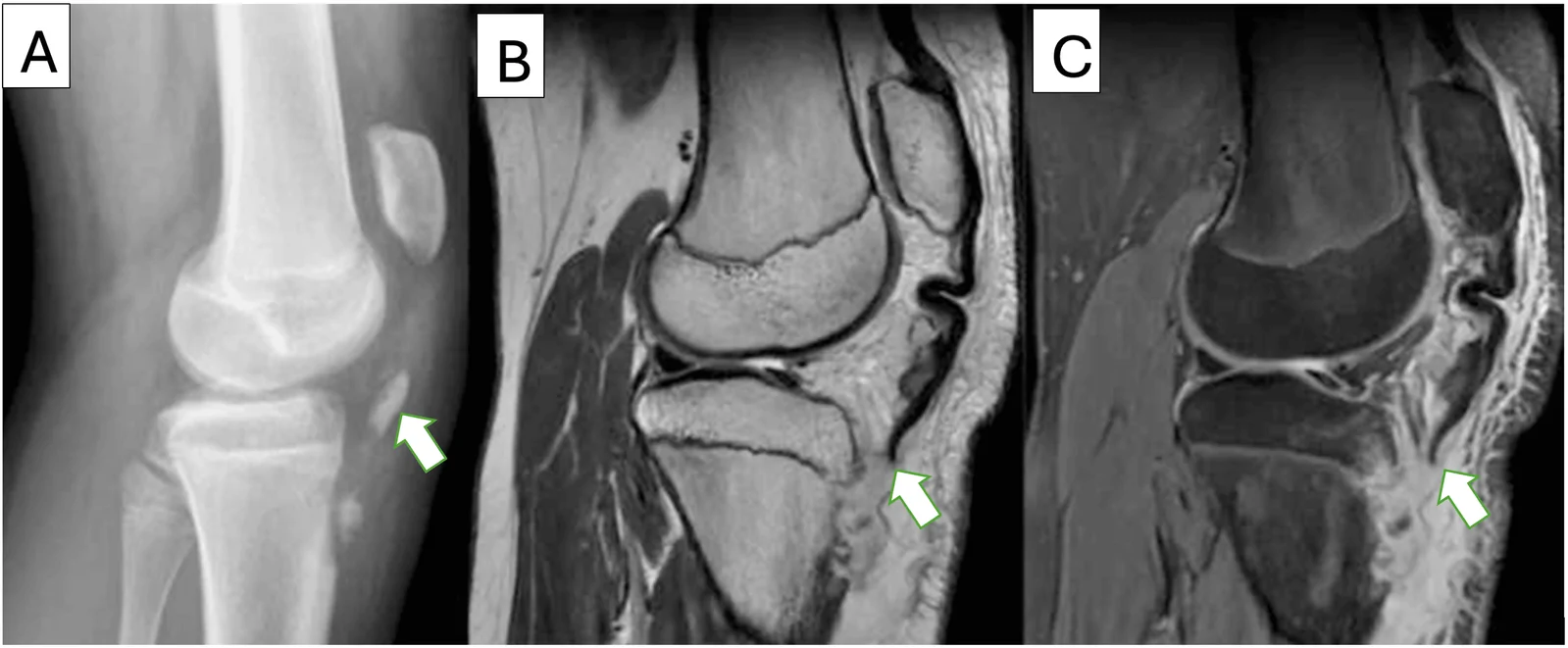
Preoperative lateral radiograph and sagittal magnetic resonance image of case 3. (A) Lateral radiograph, (B) sagittal T1-weighted MRI, and (C) sagittal fat-suppressed T2-weighted MRI demonstrating an Ogden type IIB tibial tubercle fracture without complete rotational displacement of the fragment. The distal patellar tendon avulsion (arrows) is evident on MRI despite the absence of fragment rotation, demonstrating that this radiographic sign is not universally present in bifocal extensor mechanism injuries.

**Figure 6:**
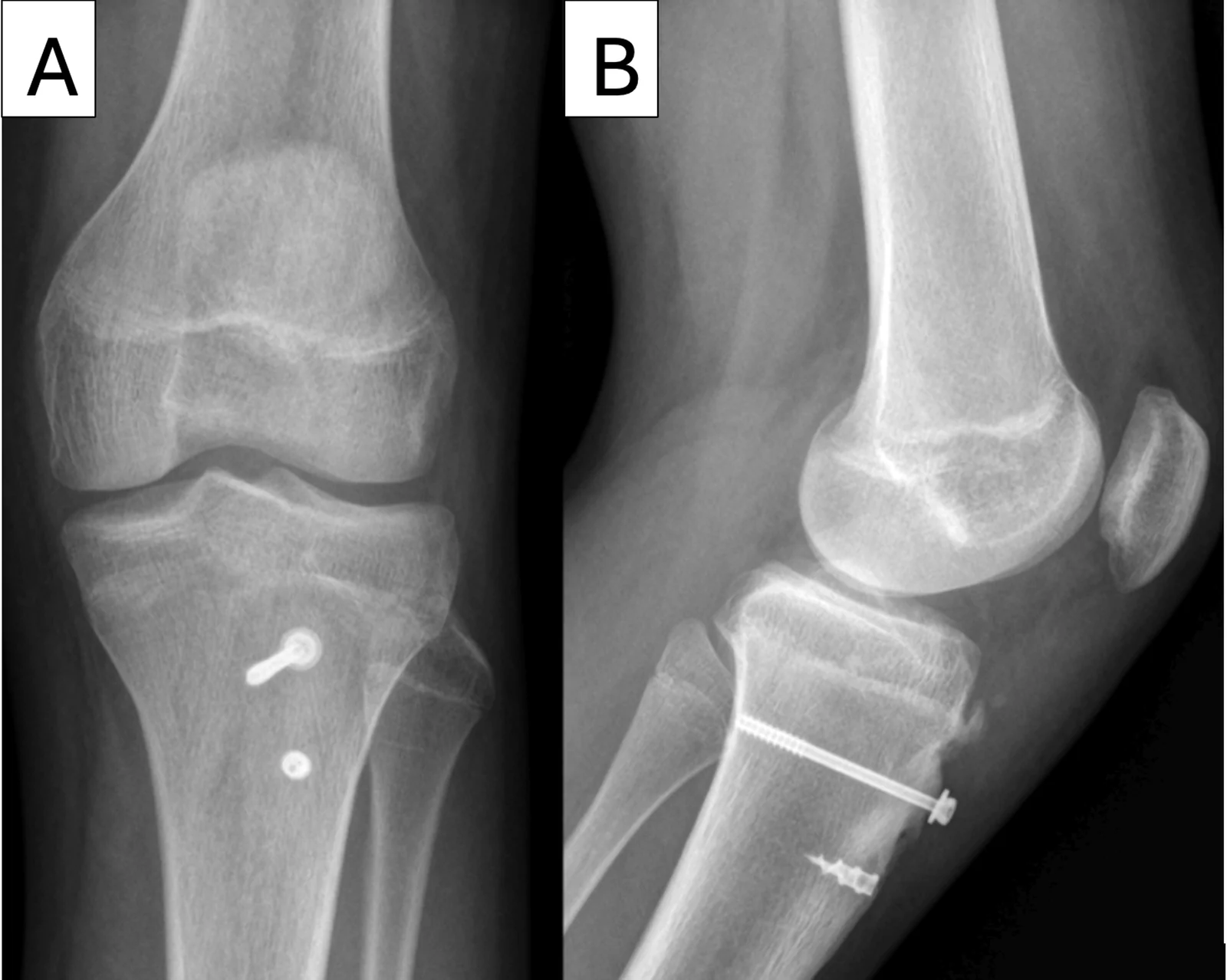
Postoperative radiographs of case 3. (A) Anteroposterior and (B) lateral radiographs obtained six months postoperatively demonstrating maintained reduction, stable fixation, and fracture union following open reduction and internal fixation of the tibial tubercle with concomitant patellar tendon repair.

Patient 3 was a 17-year-old boy who sustained a left knee injury after a ground-level fall with direct trauma to the knee. He developed pain, swelling, and functional limitation. Physical examination showed ecchymosis and increased volume around the tibial tubercle, and he was unable to activate the extensor mechanism. He had no comorbidities or antecedent apophysitis. Preoperative radiographs demonstrated an Ogden type IIB tibial tubercle fracture without fragment rotation, and magnetic resonance imaging confirmed the associated distal patellar tendon avulsion (Figure 5). The lesion consisted of an avulsion fracture of the tibial tubercle with moderate comminution and complete distal patellar tendon avulsion with an attached 17 mm osseous fragment. He underwent open reduction and internal fixation with one cannulated screw and washer, followed by tendon repair with a suture anchor and high-strength sutures using a Krackow technique. Radiographs obtained at six months postoperatively demonstrated maintained reduction and fracture union (Figure 6). At final follow-up, 14 months after surgery, he had recovered full range of motion, was asymptomatic, and had no functional limitation or complications. His Lysholm score was 99, and his Tegner score improved from 6 before injury to 7 at final follow-up. He had full active extension and reported a higher activity level than before injury.

A summary of the clinical characteristics, surgical management, and outcomes of the three patients is presented in Table 1.

**Table 1:** Summary of the clinical characteristics, surgical management, and outcomes of the three patients. Comparison of patient characteristics, fracture pattern, radiographic findings (including complete rotational displacement of the tibial tubercle fragment), surgical treatment, and clinical outcomes in the three reported cases. ORIF: open reduction and internal fixation; ROM: range of motion.

Variable	Case 1	Case 2	Case 3
Age/sex	13/Male	16/Male	17/Male
Injury mechanism	Jumping during rugby	Jumping injury	Fall with direct knee trauma
Ogden classification	IIB	IB	IIB
Key physical findings	Patella alta, inability to perform straight-leg raise	Patella alta, inability to perform straight-leg raise	Swelling/ecchymosis, extensor mechanism deficit
Radiographic clue	Complete fragment rotation	Complete fragment rotation	No fragment rotation
Patellar tendon injury	Complete distal avulsion	Complete distal avulsion	Complete distal avulsion with bony fragment
Surgical treatment	ORIF + suture anchors + Krackow repair	ORIF + suture anchors + Krackow repair	ORIF + suture anchor + Krackow repair
Clinical outcome	Union, returned to sports	Union, returned to sports	Full ROM, asymptomatic
Follow-up	20 months	27 months	14 months

## Discussion

Combined tibial tubercle fracture and patellar tendon rupture is a rare but clinically important injury pattern in adolescents. Available literature remains limited to isolated reports and small series [2,3,6-11]. In our series, all three patients were adolescent boys with acute extensor mechanism insufficiency, and two showed complete rotational displacement of the tibial tubercle fragment on the initial radiograph.

A plausible mechanism is a sudden eccentric quadriceps contraction against a flexed knee or fixed foot, producing avulsion of the tibial tubercle in the immature skeleton [3]. When the fragment rotates while remaining partially constrained by soft tissues, it may act as a fulcrum that increases tension on the distal patellar tendon and contributes to rupture. This concept is consistent with the pattern observed in our first two cases and with prior reports of bifocal extensor mechanism injury [2,5].

From a diagnostic standpoint, our findings support careful assessment of standard radiographs. In two patients, complete fragment rotation was visible on the initial lateral radiograph and raised suspicion for associated patellar tendon injury. However, because this finding was absent in our third patient and no comparison group was available, complete fragment rotation should be considered a potential radiographic clue rather than a validated diagnostic sign. Larger studies are needed to determine its diagnostic accuracy. When uncertainty persists, advanced imaging may still help define the full extent of soft-tissue injury and assist with surgical planning [2,5].

There is no universally accepted fixation construct for this lesion. Reported options include transosseous sutures, screw fixation, suture anchors, and augmentation in selected cases [4,7,8]. In our series, all patients underwent single-stage surgical treatment addressing both the osseous and tendinous components. Although fixation details varied among cases, all three patients recovered full active extension, had high Lysholm scores, and returned to their preinjury or higher activity level without complications.

Our findings are consistent with previous case reports and small case series describing favorable outcomes after prompt recognition and single-stage surgical repair of both the osseous and tendinous injuries [2,6-11]. Similar to prior reports, all patients in our series recovered full active knee extension and returned to their previous level of activity. The present series also highlights complete rotational displacement of the tibial tubercle fragment as a potentially useful radiographic clue that warrants further investigation in larger comparative studies.

This study has limitations. The cohort is small, reflecting the rarity of the condition, and the study is retrospective and descriptive. Nonetheless, all three patients had follow-up beyond one year, ranging from 14 to 27 months, which strengthens assessment of early functional recovery.

## Conclusions

Combined tibial tubercle fracture and patellar tendon rupture is an uncommon bifocal extensor mechanism injury in adolescents. Complete rotational displacement of the tibial tubercle fragment on the lateral radiograph may serve as an early diagnostic clue to associated patellar tendon injury, although its absence does not exclude the diagnosis. Single-stage surgical treatment addressing both the osseous and tendinous components restored active extension and was associated with excellent functional outcomes in this series.
